# Who Are the Young People Choosing Web-based Mental Health Support? Findings From the Implementation of Australia's National Web-based Youth Mental Health Service, eheadspace

**DOI:** 10.2196/mental.5988

**Published:** 2016-08-25

**Authors:** Debra Rickwood, Marianne Webb, Vanessa Kennedy, Nic Telford

**Affiliations:** ^1^ headspace National Youth Mental Health Foundation Melbourne Australia; ^2^ University of Canberra Canberra Australia

**Keywords:** mental health, adolescent, help-seeking behavior, telemedicine, counseling, Internet

## Abstract

**Background:**

The adolescent and early adult years are periods of peak prevalence and incidence for most mental disorders. Despite the rapid expansion of Web-based mental health care, and increasing evidence of its effectiveness, there is little research investigating the characteristics of young people who access Web-based mental health care. headspace, Australia’s national youth mental health foundation, is ideally placed to explore differences between young people who seek Web-based mental health care and in-person mental health care as it offers both service modes for young people, and collects corresponding data from each service type.

**Objective:**

The objective of this study was to provide a comprehensive profile of young people seeking Web-based mental health care through eheadspace (the headspace Web-based counseling platform), and to compare this with the profile of those accessing help in-person through a headspace center.

**Methods:**

Demographic and clinical presentation data were collected from all eheadspace clients aged 12 to 25 years (the headspace target age range) who received their first counseling session between November 1, 2014 and April 30, 2015 via online chat or email (n=3414). These Web-based clients were compared with all headspace clients aged 12 to 25 who received their first center-based counseling service between October 1, 2014 and March 31, 2015 (n=20,015).

**Results:**

More eheadspace than headspace center clients were female (78.1% compared with 59.1%), and they tended to be older. A higher percentage of eheadspace clients presented with high or very high levels of psychological distress (86.6% compared with 73.2%), but they were at an earlier stage of illness on other indicators of clinical presentation compared with center clients.

**Conclusions:**

The findings of this study suggest that eheadspace is reaching a unique client group who may not otherwise seek help or who might wait longer before seeking help if in-person mental health support was their only option. Web-based support can lead young people to seek help at an earlier stage of illness and appears to be an important component in a stepped continuum of mental health care.

## Introduction

The adolescent and early adult years are periods of peak prevalence and incidence for most mental disorders. One in 4 young people will experience a clinically relevant mental health problem within any 12-month period, with 75% of all mental disorders emerging before 25 years of age [1]. These disorders can have a wide range of major adverse effects on a young person’s quality of life, impacting relationships with family and friends, educational attainment, and future economic stability [2].

The high prevalence of mental disorder in young people is not matched by a commensurate level of mental health service use. Rather, there is a marked mismatch between the prevalence of disorder and professional help-seeking [3]. In Australia, this mismatch is greatest for 16- to 24-year-old males. The 2007 National Survey of Mental Health and Wellbeing (NSMHW) found that only 13% of males in this age range who had experienced a mental disorder in the previous 12 months sought professional help [4]. Among females, the NSMHW found that 31.2% of the 16 to 24 age bracket who had experienced a mental disorder in the previous 12 months had sought help for a mental disorder [4].

Even for those young people who do seek help, there is often a considerable delay between onset of symptoms and accessing services. This varies according to factors such as type of disorder, gender, population group, and geographical location [5]. Seeking professional help in an appropriate and timely manner can reduce the long-term impact of many mental health difficulties [3], while delays in accessing help have been shown to have a significant impact on social, educational, and vocational outcomes for young people [6].

The monetary costs to the Australian economy associated with untreated mental disorders in young people aged 12 to 25 have been estimated at more than AUD$10.6 billion annually (equivalent to US$8.6 billion) [7], due to costs associated with unemployment, absenteeism, and welfare payments [8]. The World Economic Forum expects that costs of mental illness will double over the next 20 years worldwide [9], which highlights the need to develop and implement services and approaches that can effectively engage young people in appropriate and timely help-seeking [10].

The reasons young people do not access mental health services in accordance with their level of need are complex. They include: stigma (which includes embarrassment and concern about what others think); negative attitudes to and poor past experiences of treatment; problems recognizing symptoms; lack of awareness of available services; confidentiality concerns; and a preference for self-reliance or drawing on nonprofessional support through family or friends [11]. In addition to these personal factors, a number of structural barriers exist, including location, cost, and availability of services [12].

Given these substantial barriers to seeking help in-person, it is not surprising that many young people, including those with a probable serious mental illness, are turning to the Internet for information about mental health issues [13]. The Internet is an appealing alternative to traditional in-person services due to its accessibility, interactivity, and anonymity; it offers a wide variety of health information that is not impacted by structural constraints [14].

In response to demand for Web-based mental health information and support, and in order to address barriers associated with in-person help-seeking, Web-based options are rapidly being developed to enable young people to access information, support, and mental health interventions via communication technologies [15]. The effectiveness of Web-based counseling is a growing research area. A systematic review found that Web-based counseling was effective, despite the absence of face-to-face cues and often slow pace of sessions [16]. Young people report feeling safe and less emotionally exposed using Web-based counseling compared with in-person or telephone counseling [17]. There is also evidence to suggest that Web-based counseling can result in a similar level of impact [18], client satisfaction, and therapeutic alliance [19] compared with counseling conducted in-person.

There has been a concerted effort in Australia to make mental health counseling widely available and accessible to young people. In 2006, the Australian Federal Government established headspace, the National Youth Mental Health Foundation [20]–an enhanced primary care model for youth mental health care [21]. headspace centers have been progressively rolled out across Australia, and will soon reach 100 centers nation-wide. These centers are designed to break down common barriers to help-seeking and enable young people early access to in-person mental health counseling and support.

To extend the reach of headspace and further enable access for young people who do not live near a headspace center or do not want to visit one in person, eheadspace [22] was developed as a clinically supervised, youth-friendly, confidential, and free Web-based mental health support and information service. eheadspace commenced as a pilot in July 2010 and was rolled out nationally in July 2011. It offers synchronous online chat, asynchronous email, and telephone-based mental health counseling to young people aged 12 to 25 Australia-wide.

Despite the rapid expansion of Web-based mental health care options, supporting evidence is still emerging and there is little research investigating the characteristics of young people accessing Web-based counseling. The current study addresses this gap by presenting the first comprehensive profile of young people who access Web-based counseling (via eheadspace) and comparing them with those who access in-person counseling (via headspace centers). headspace is ideally placed to explore differences between Web-based and in-person clients as it offers both service modes and collects corresponding data from young people accessing each service type.

## Methods

### Participants and Procedure

Participants were all eheadspace clients aged 12 to 25 years (the headspace target age range) who received their first counseling session during the 6-month period November 1, 2014 to April 30, 2015 via online chat or email (n=3414). Clients who received their first counseling session via the phone were not included given the focus of this study is young people who choose to seek Web-based help.

These Web-based clients were compared with all headspace clients aged 12 to 25 who received their first center-based counseling session in a similar 6-month period (October 1, 2014 to March 31, 2015) (n=20,015 clients from across 81 centers). headspace center clients have previously been described in Rickwood et al [21].

headspace implements a minimum dataset across centers and eheadspace. Part of the minimum dataset is completed by the young person accessing counseling, while another section is completed by their service provider. While data items are completed at every occasion of service, this study examines first-time data recorded at initial presentation.

The data from both young people and service providers are collected via electronic forms. Data are de-identified via encryption and extracted to the headspace national office data warehouse. All headspace clients (eheadspace and center), agree to various terms and conditions including that the data they provide are used at an aggregate level to evaluate, report on, and improve headspace services.

Ethics approval was obtained through quality assurance processes, comprising initial consideration and approval by the headspace Clinical, Research, and Evaluation Committee, and subsequent consideration and approval by the headspace Board of Directors. The consent processes were reviewed and endorsed by an independent body, Australasian Human Research Ethics Consultancy Services.

### Measures

Demographic measures reported comprise: age in years; gender; Aboriginal and Torres Strait Islander background; country of birth; living situation; location; and work and study situation. Client clinical presentation was measured by self-reported reason for presentation, level of psychological distress as measured by the 10-item Kessler Psychological Distress Scale (K10) [23], and days out of role [24]. Service provider–rated items include: stage of illness using the categories of no mental disorder, mild to moderate symptoms, subthreshold symptoms not reaching full diagnosis, diagnosed disorder, periods of remission, or serious and ongoing disorder without periods of remission [25]; and overall functioning using the Social and Occupational Functioning Assessment Scale [26].

### Analyses

Descriptive statistics are presented, primarily percentages of young people according to presenting characteristics by mode of service (eheadspace vs centers). National population data comparisons are provided, where available. Pearson’s chi-square tests of contingencies were undertaken to explore whether being an eheadspace compared with a headspace center client was associated with presenting characteristics. Effect sizes are reported as Phi or Cramer’s *V*, with magnitude based on Cohen [27].

## Results

### Demographic Characteristics

Table 1 provides information about demographic characteristics of eheadspace and center clients, and where possible national data are provided as a comparison. More eheadspace than headspace center clients were female, and more eheadspace than headspace center clients were transgender, transsexual, intersex, or another gender. In contrast, more than one-third of center clients were male compared with less than one-fifth of eheadspace clients. The association between gender and type of service (eheadspace or headspace center) was significant (χ^2^_1_=598.7, *P<*.001), but quite small, Cramer’s *V*=.17.

The peak age of presentation for eheadspace was the same as that for centers (15-17 years of age). Slightly more eheadspace clients than center clients were in the 15 to 17 and 18 to 20 age brackets. Much fewer eheadspace than center clients were aged 12 to 14, while more eheadspace than center clients were aged 21 to 25. The association between age and type of service was significant (χ^2^_1_=317.5, *P<*.001), but small, Cramer’s *V*=.12.

A lower percentage of eheadspace than center clients identified as Aboriginal or Torres Strait Islander; the association was significant (χ^2^_1_=123.4, *P<*.001), but small, φ = .08. A higher percentage of eheadspace than center clients reported that they were born outside Australia, and the association was significant (χ^2^_1_=24.2, *P<*.001), but very small, φ = .03. In line with population trends, the most common places of birth outside Australia for both eheadspace and center clients were in the United Kingdom and New Zealand. There were 96% of eheadspace clients who reported that they did not speak a language other than English at home. This compares with 92.8% of center clients and 80.3% of the general population aged over 5 years [31]. The association between language spoken at home (English or not English) and type of service was significant (χ^2^_1_=56.5, *P<*.001), but very small, φ = .05.

The location of eheadspace clients according to the 2011 edition of the Australian Statistical Geography Standard was largely in line with the location of the Australian population as estimated in 2014 [32]. More eheadspace than center clients were from major cities and outer regional areas and fewer were from inner regional and remote areas. The location of center clients is dependent on the location of centers as most clients live within 10 km of the center they attend [29]. The association between location and type of service was significant (χ^2^_1_=58.2, *P<*.001), but small, Cramer’s *V*=.05.

More eheadspace than headspace center clients indicated that they had stable accommodation. A slightly lower percentage of eheadspace than center clients reported that accommodation was an issue, they were at risk of being homeless, or that they were currently homeless. This compares with 2011 Census estimates that 0.7% of the Australian population aged 12 to 24 years were homeless or in marginal housing [33]. The association between living situation (in stable accommodation or not in stable accommodation) and type of service was significant (χ^2^_1_=2002.0, *P<*.001), and of medium strength, φ = .31.

A higher percentage of eheadspace than center clients indicated that they were currently at school (55.2% compared with 49.3%), while a similar percentage of eheadspace and center clients indicated they were currently engaged in higher education (18.3% compared with 18.8%). The association between education level (at school or in higher education) and type of service was significant (χ^2^_1_=7.4, *P=*.006), but very small, φ = .02. Among those aged 18 to 25 years, a lower percentage of eheadspace than center clients were not engaged in employment, education, or training. The association between not working or studying and type of service was significant (χ^2^_1_=105.9, *P<*.001), but small, φ = .10.

**Table 1:** Demographic characteristics of eheadspace and headspace center clients with national comparison data.

Demographic characteristics		eheadspace (%)	headspace centers (%)	National (%)
**Gender**				
	Female	78.1	59.1	48.7^a^
	Male	18.9	39.9	51.3^a^
	Transgender, transsexual, intersex, or another gender	3.0	1.0	Not available
**Age group**				
	12-14	10.2	23.6	31.2 (10-14)^a^
	15-17	36.8	33.1	
	18-20	28.8	22.9	32.4 (15-19)^a^
	21-25	24.2	20.4	36.4 (20-24)^a^
**Aboriginal/Torres Strait Islander**		3.2	8.8	3.7^b^
**Born outside Australia**		10.3	7.8	17.0^c^
**Only speak English at home**		96.0	92.8	80.3^d^
**Location**				
	Major city	70.2	65.1	70.9^e^
	Inner regional	21.4	26.3	18.1^e^
	Outer regional	7.5	6.7	8.8^e^
	Remote or very remote	0.9	1.9	2.2^e^
**Living situation**				
	Stable	90.4	89.1	Not available
	An issue	8.4	8.8	Not available
	At risk	1.1	1.6	Not available
	Homeless	0.1	0.5	0.7^f^
**Not engaged in education,** **employment, or training**		15.6^g^	27.2^g^	27.3^h^

^a^10-24 years [28].

^b^12-25 years [29].

^c^10-24 years [30].

^d^5 years and older [31].

^e^All ages [32].

^f^12-24 years [33].

^g^18-25 years.

^h^17-24 years [34].

**Table 2:** Clinical presentation characteristics of eheadspace and headspace center clients.

Presenting characteristics		eheadspace (%)	headspace centers (%)
**Reason for contact**			
	Problems with how they felt	79.3	75.9
	Relationship problems	13.6	11.0
	Physical health issues	1.6	2.1
	School or work problems	3.6	7.8
	Alcohol or other drug problems	1.6	2.4
	Vocational concerns/assistance	0.3	0.8
**High/very high psychological distress**		86.6	73.2
**Stage of illness**			
	No mental disorder	27.5	15.7
	Mild/moderate symptoms	53.1	43.0
	Subthreshold	13.8	19.1
	Threshold diagnosis	4.9	16.3
	Remission	0.5	1.4
	Serious, ongoing	0.2	4.5
**Not previously seen by a mental health professional**		46.4	44.1
**Days out of role**			
	None	34.7	41.2
	1-3 days	30.0	26.3
	4-6 days	15.3	13.1
	7-9 days	14.9	5.6
	10+ days	5.1	13.8
**Serious or major impairment in functioning**		5.0	11.7

### Clinical Presentation Characteristics

Table 2 provides information about the clinical presentation characteristics of eheadspace and center clients. Approximately three-quarters of both eheadspace and center clients indicated that their primary reason for seeking help was regarding problems with how they felt. Specifically, 45.3% of eheadspace clients indicated that they were feeling sad or depressed, and 15.3% indicated that they were feeling anxious.

The second most reported reason for seeking help (for both eheadspace and center clients) was for relationship problems. Fewer eheadspace than center clients indicated that their primary reason for seeking help was physical health issues, school/work problems, alcohol or other drug problems, or vocational concerns. The association between reason for contact and type of service was significant (χ^2^_1_=2619.7, *P<*.001), and of medium strength, Cramer’s *V*=.37.

Across both services the majority of clients presented with high or very high levels of psychological distress, although the percentage of eheadspace clients was higher than the percentage of center clients. Comparatively, the 2007 NSMHW data [4] indicated that 9% of those in the general community, and 21% of young people diagnosed with a mental disorder, aged 16 to 24 had high or very high levels of psychological distress. The association between psychological distress and type of service was significant (χ^2^_1_=3277.1, *P<*.001), and of medium strength, φ = .40.

**Figure 1:**
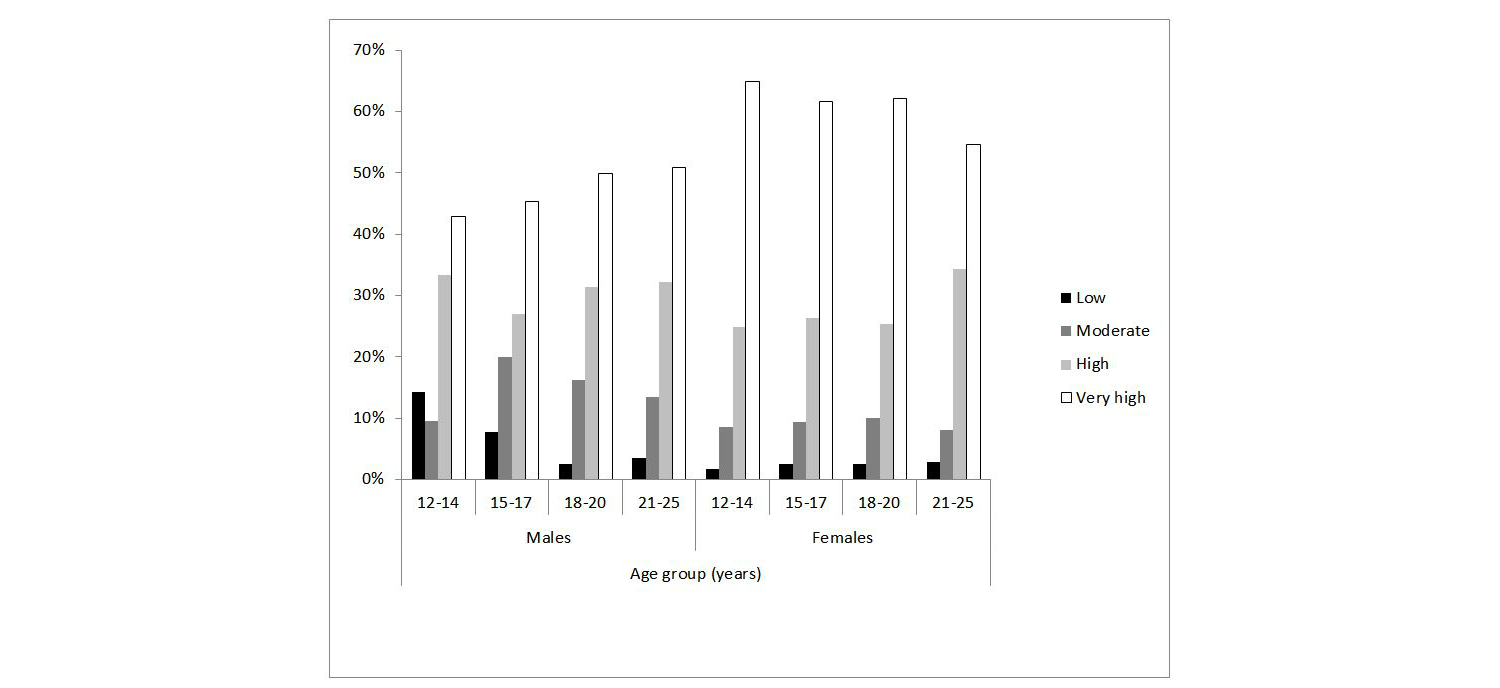
Percentage of eheadspace clients at each level of psychological distress, by age group and gender (males and females only).

**Figure 2:**
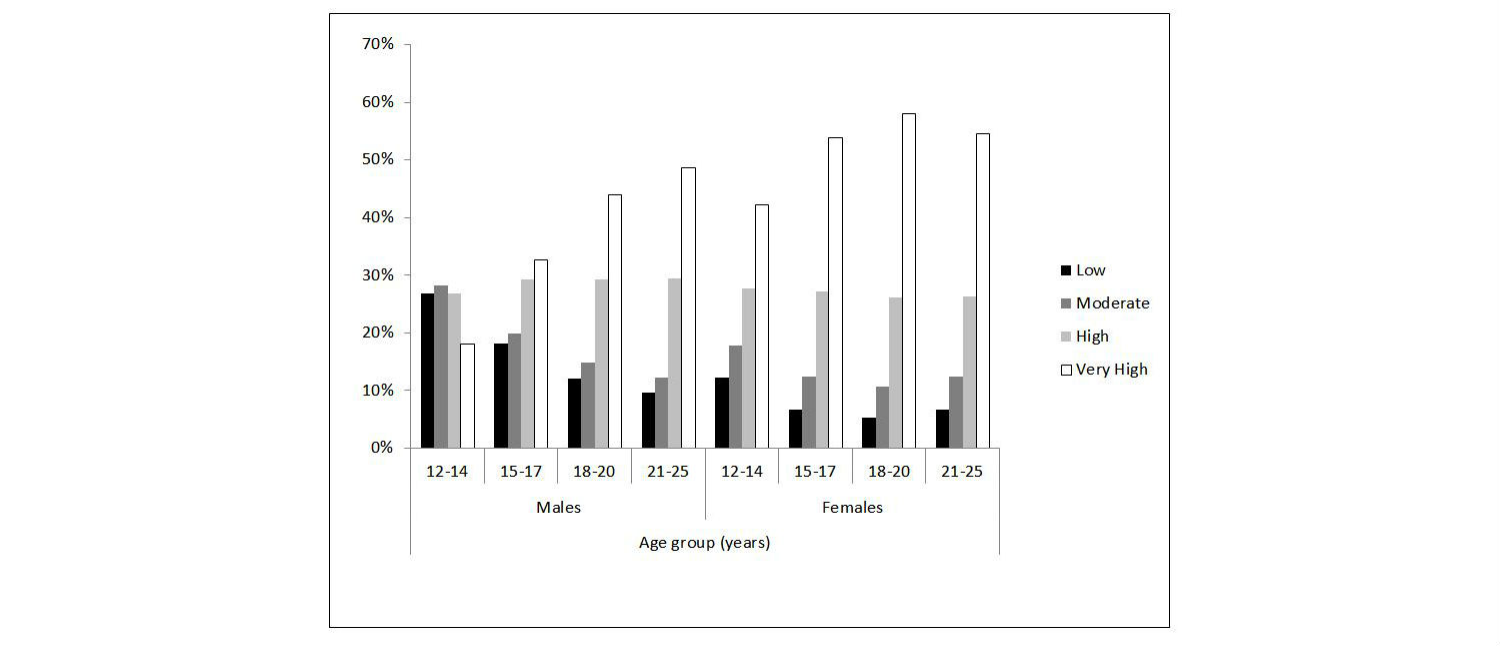
Percentage of headspace center clients at each level of psychological distress, by age group and gender (males and females only).

Figure 1 displays the percentage of eheadspace clients that presented with high or very high levels of psychological distress broken down by age and gender while Figure 2 does the same for center clients. As shown, a higher percentage of eheadspace than center clients presented with very high distress–this was the case for both genders and all age groups, particularly 12- to 14-year-old males. For both eheadspace and centers, a higher percentage of females than males reported very high levels of psychological distress across all age brackets.

Stage of illness, as estimated by service providers, indicated that more eheadspace clients than center clients presented without a mental disorder or with mild to moderate symptoms. Fewer eheadspace than center clients presented with subthreshold diagnosis, full-threshold diagnosis, periods of remission, or serious and ongoing mental disorder. Importantly, for 46.1% of eheadspace clients and 16.1% of center clients, clinicians recorded that they did not have enough information available to make an assessment of stage of illness and these clients were excluded from these comparisons. The association between stage of illness and type of service was significant (χ^2^_1_=431.7, *P<*.001), but quite small, Cramer’s *V*=.15.

A higher percentage of eheadspace than center clients reported that they had never seen a mental health professional prior to their eheadspace/center visit. The association between prior help-seeking and type of service was significant (χ^2^_1_=5.9, *P=*.015), but small, φ = .02. Fewer eheadspace than center clients reported that they had been able to carry out their usual activities every day in the last 2 weeks. More eheadspace than center clients reported that they had 1 to 3, 4 to 6, or 7 to 9 days out of role, and fewer eheadspace than center clients reported they had 10 or more days out of role. The association between days out of role and type of service was significant (χ^2^_1_=563.6, *P<*.001), but quite small, Cramer’s *V*=.17.

Social and vocational functioning scores as assessed by service providers indicated that a lower percentage of eheadspace than center clients experienced serious or major impairment. The association between having a serious or major impairment and type of service was significant (χ^2^_1_=93.03, *P<*.001), but small, φ = .07. Importantly, for 33.5% of eheadspace clients and 8.3% of center clients, clinicians recorded that they did not have enough information available to make an assessment of social and occupational functioning and these clients were excluded from these comparisons.

## Discussion

### Key Results

These are the first data to compare the characteristics of young people seeking Web-based mental health counseling and in-person mental health counseling through the headspace service system. headspace is specifically designed to break down the barriers to young people accessing mental health support and the same branding and service promotion is applied to both the Web-based and in-person counseling services. While many similarities were observed between the two groups of clients, important differences were identified.

The most striking finding was the extent of preference by females for Web-based counseling compared with males―close to 80% of the Web-based clients were female. Research from other Web-based services consistently reports a similar gender effect [17,35]. National surveys indicate slightly more females than males experience mental disorder (30% compared with 23%) and that the 16- to 24-year-old age bracket has the highest disparity between females and males in seeking professional help for mental health issues (with females more than twice as likely as males to use services). However, there is clearly something about the Web-based space that particularly appeals to females as opposed to males given the gender disparity for using the Web-based space is even greater. Males were comparatively more likely to access centers, particularly the adolescent males.

In general, males are more likely to be influenced by others, particularly family, to attend mental health services [36], and this personal encouragement may be more effective at getting young men to in-person services. It is also increasingly well established that the initial engagement of young men is challenging, and requires a concerted focus on rapport building, including greater flexibility and choice in how the service is accessed, and service promotion that assertively reaches out into the spaces that young men are likely to inhabit [37]. Such engagement may be easier face-to-face, as good interpersonal communication skills can be effectively applied, by both the family members encouraging young men to seek help and the service providers building rapport. Web-based service use is more dependent on self-motivation, and engagement and rapport take more effort to build via the Internet, which may act against young men’s uptake.

For both service types there was the same peak age of presentation at 15 to 17 years, which coincides with the period when the common mental health problems of depression and anxiety develop [1]. The youngest adolescents were less than half as likely to use the Web-based service, however, possibly reflecting more restricted or closely monitored Internet access in the early teen years, as well as greater parental involvement in mental health care [34].

Young people from Aboriginal and Torres Strait Islander backgrounds were less likely to use the Web-based than the in-person service. The percentage of Aboriginal or Torres Strait Islander clients who accessed the in-person service was higher than the percentage of all Australians aged 12 to 25 years who identify as Aboriginal or Torres Strait Islander as indicated in the 2011 census [38]. Young people from culturally and linguistically diverse backgrounds were underrepresented in both service types. headspace has had a health promotion focus on young people who are Aboriginal and Torres Strait Islander through its Yarn Safe campaign [39]. This campaign commenced after the period during which the data for our study were collected. More recent data collected since this campaign indicate an increase in the number of Aboriginal and Torres Strait Islander young people to both centers and eheadspace, suggesting that targeted promotion to this community is a worthwhile investment [40].

The geographical dispersion of center clients reflects the location of centers, which have been set up across Australia to meet community needs. The external evaluation of headspace centers noted a strong relationship between the use of headspace centers and the distance of the center from a client's home, with the majority of clients living within a 10-km radius [29]. The fact that the distribution of eheadspace clients reflects the general population distribution shows that the Web-based counseling option is equitably accessible throughout Australia, but indicates that greater targeted promotion may be required in regional and remote areas.

The psychological distress results reveal that young people who use Web-based services are highly distressed, more so than when they present to in-person counseling services, but that they are also earlier in the development of a mental health problem, being at an earlier stage of illness and less likely to have previously accessed mental health care. These results are an important validation of Web-based access as part of stepped-care approaches, revealing that this modality does enable earlier access. Nevertheless, even with earlier presentations, young people are still highly distressed by their symptoms and this distress needs to be a major focus of the initial Web-based counseling response [41].

Distress is likely to be greater for Web-based clients because service use is closer in time to the symptoms that are distressing. Clients of in-person headspace counseling services have to wait from when they make an appointment to when they receive their service [37]; so, in the Web-based environment service use is more proximal to distressing symptoms and events. Interpreted this way, the high psychological distress scores reported by Web-based clients can be taken as evidence that Web-based clients are able to access help at the time they most need it–that is, when they are most affected by their issues.

The issues that Web-based clients seek help for are also more strongly related to current feelings of depression and anxiety, as well as relationship problems. In contrast, clients of the in-person headspace centers are accessing a wider range of health care options, including for physical health issues. Again, this supports the value of Web-based counselling in addressing current emotional distress─there is clearly a need for this type of support, especially for teenage girls. The Young Minds Matter Australian national survey of young people’s mental health and wellbeing reported high levels of major depressive disorder, psychological distress, self-harm, and suicidal behaviors among adolescents [42]. The results of this most recent survey revealed an alarming picture of distress, especially for teenage girls aged 16 and 17, with 19.6% experiencing major depressive disorder, 22.8% reporting self-harm, and 15.4% seriously considering attempting suicide. That eheadspace seems to be most effectively reaching this at-risk demographic group is a very positive response for Australia.

The Web-based clients were more likely to be living in stable accommodation, which may suggest that Web-based counseling access is easier for those at home with a computer and Internet connection, and it may be more difficult for young people to go on the Internet in other living situations. While surveys show that almost all young people in Australia have Internet access, for young people who are homeless or couch surfing this can be through public facilities like libraries or drop in centers, or through use of a friend’s computer [43], which may not be conducive to engaging in something as personal and time consuming as counseling [44].

### Limitations and Further Research

This study has a number of limitations, including the diminishing sample size for eheadspace when broken down by age and gender categories, despite the overall very large sample sizes. The sample for eheadspace was particularly small for some variables, such as stage of illness and psychosocial functioning, with Web-based clinicians having more difficulty making these judgements during first presentation. Better guidance around these issues may be required in order to improve clinicians’ ability to make these assessments.

It is important to acknowledge the possibility that some clients may use both health service types. Unique client codes are used in each service, however, a question in the center dataset asks young people if they have accessed eheadspace, and 5.6% indicated they had. It would be of interest to explore whether clients who access Web-based counseling or in-person counseling exclusively differ from those who access both types, and this is something that could be explored in future research.

Despite these limitations, the study represents an important step in understanding young people who access Web-based counseling. Future research and analysis should investigate the types of interventions that eheadspace clients are receiving and determine whether the approach is making a difference to their mental health and wellbeing. While Web-based counseling certainly has a role in the mental health care continuum, more research is needed to determine how it can best be used to improve access and engage hard to reach young people, as well as its role in stepped care and collaborative approaches between Web-based and in-person services.

### Conclusions

During a period when mental health programs and services are being reviewed in Australia [45], it is timely to investigate the young people presenting to Web-based counseling and determine whether they differ substantively from those attending in-person services. The findings of this study suggest that the eheadspace Web-based service is reaching a unique client group who may not otherwise seek help or who might wait longer before seeking help if in-person support was their only option. In particular, Web-based support was shown to be highly accessed by young females with depressive symptoms, which is a demographic group that is growing and has been identified as particularly vulnerable and in need of greater focus. Web-based support can lead young people to seek help at an earlier stage of illness and is appealing to young people who have never sought mental health assistance before. This is important to enable young people to access support at the earliest opportunity with the aim of reducing the likelihood of more serious mental health problems developing.

## Abbreviations

NSMHW: National Survey of Mental Health and Wellbeing
